# Evaluating vegetation effects on animal demographics: the role of plant phenology and sampling bias

**DOI:** 10.1002/ece3.2148

**Published:** 2016-04-24

**Authors:** Daniel Gibson, Erik J. Blomberg, James S. Sedinger

**Affiliations:** ^1^ Program in Ecology, Evolution and Conservation Biology Department of Natural Resources and Environmental Science University of Nevada Reno Mail Stop 186 Reno Nevada 89557 USA; ^2^ Department of Fish and Wildlife Conservation Virginia Polytechnic Institute and State University Blacksburg Virginia 24060 USA; ^3^ Department of Wildlife, Fisheries, and Conservation Biology University of Maine 5755 Nutting Hall Room 210 Orono Maine 04469 USA

**Keywords:** Grass height, nest survival, plant phenology, sampling bias

## Abstract

Plant phenological processes produce temporal variation in the height and cover of vegetation. Key aspects of animal life cycles, such as reproduction, often coincide with the growing season and therefore may inherently covary with plant growth. When evaluating the influence of vegetation variables on demographic rates, the decision about when to measure vegetation relative to the timing of demographic events is important to avoid confounding between the demographic rate of interest and vegetation covariates. Such confounding could bias estimated effect sizes or produce results that are entirely spurious. We investigated how the timing of vegetation sampling affected the modeled relationship between vegetation structure and nest survival of greater sage‐grouse (*Centrocercus urophasianus*), using both simulated and observational data. We used the height of live grasses surrounding nests as an explanatory covariate, and analyzed its effect on daily nest survival. We compared results between models that included grass height measured at the time of nest fate (hatch or failure) with models where grass height was measured on a standardized date – that of predicted hatch date. Parameters linking grass height to nest survival based on measurements at nest fate produced more competitive models, but slope coefficients of grass height effects were biased high relative to truth in simulated scenarios. In contrast, measurements taken at predicted hatch date accurately predicted the influence of grass height on nest survival. Observational data produced similar results. Our results demonstrate the importance of properly considering confounding between demographic traits and plant phenology. Not doing so can produce results that are plausible, but ultimately inaccurate.

## Introduction

For many animals, vegetation represents an important habitat feature, and thus as a component of the environment plays a critical role in affecting both ecological and evolutionary processes. For this reason, understanding how vegetation structure and composition affect animal demographics, individual fitness, and population growth, is a key to both basic and applied research. Modern methods of demographic analysis (e.g., White and Burnham 1999; Dinsmore et al. 2002; Kery and Schaub 2012) have substantially improved our ability to understand the influence of vegetation and other environmental variables on demographic rates.

Many demographic analyses are ultimately rooted in regression‐based techniques, where a response variable (the demographic rate) is explained as a function of one or more predictor terms (e.g., a vegetation covariate) based on a modeled relationship (e.g., a logistic regression). Various criteria, such as *P*‐values, AIC scores, or credible intervals, are used to establish whether the modeled relationship has statistical support, which in turn is presumed to indicate a true biological relationship between the vegetation metric and the demographic rate. Central to this conclusion is an implicit assumption that the covariance between the covariate and response variable is driven by the true underlying mechanism, and not by sampling bias associated with the design of data collection. In the case of a relationship between an environmental variable (e.g., vegetation height) and a demographic rate, we therefore assume that a positive or negative association between the two was driven by the influence of vegetation on the demographic rate, and not by a spurious correlation caused by measurement error. Such error is often associated with imprecision of an instrument or operator, but it can also be influenced by selection bias and preferential sampling (Diggle et al. 2010), which may lead to biased parameter estimates (Muff et al. 2015).

The implications of random sampling error for model convergence, fit, and accuracy have been discussed thoroughly in general (Anderson and Gerbing 1984) and in ecological scenarios specifically (Walters and Ludwig 1981; Ostermiller and Hawkins 2004; Staples et al. 2004). These efforts collectively suggest that most issues regarding random sampling error can be largely ameliorated through increased sample sizes. In contrast, directional (nonrandom) sampling error is predominantly ignored in ecological contexts but can substantially bias results. This may be particularly true for longitudinal data where loss of individuals through time (e.g., through mortality) may affect the distribution of the remaining sampled population (Alexander et al. 1986; Goodman and Blum 1996). More importantly, spurious relationships can be observed between measured variables and demographic rates if individual sampling is somehow associated with the individual's attrition (i.e., how long an individual remains in the sample; Goodman and Blum 1996).

**Figure 1:**
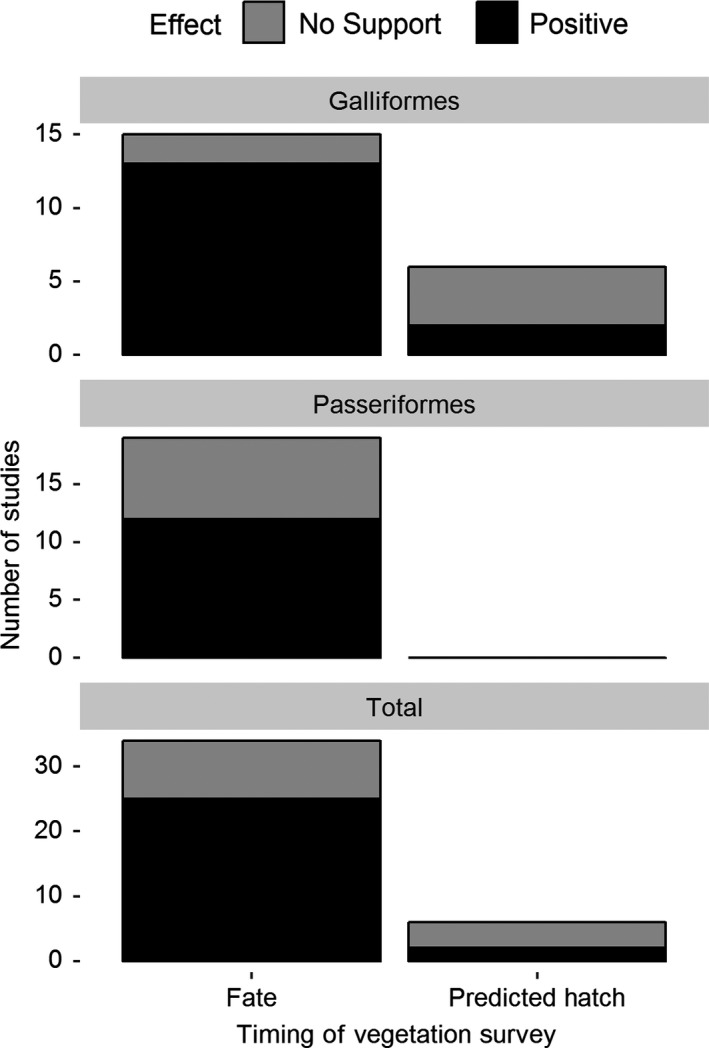
Summary of literature review assessing variation in study design for studies measuring nest site vegetation for Galliformes (top panel) and Passeriformes (center panel) in grasslands and shrublands. Two common survey protocols included sampling nest vegetation at nest fate (i.e., hatch or failure) or on a predicted hatch date, and publications reported positive (black) and no support for an effect (gray) of grass.

During the growing season, plant phenology produces temporal variation in vegetation composition, structure, and total biomass. For many animals, key aspects of the annual life cycle often coincide with the growing season (Jones and Cresswell 2010; Miller‐Rushing et al. 2010), and are therefore inherently coincident with plant growth. In birds, nesting represents the central element of annual reproduction, and parents often time nesting to produce young at the height of the growing season when food resources are most abundant (Nussey et al. 2005). In studies of avian nesting ecology, investigators frequently measure a suite of vegetation features to provide covariates for nest survival analysis (e.g., Pitman et al. 2005; Gregory et al. 2011; Davis et al. 2014). Typically vegetation is sampled subsequent to nest fate (success or failure) to minimize disturbance at active nests. However, there is inconsistency among studies with respect to how successful and failed nests are sampled; some investigators elect to sample vegetation at or near the timing of fate, whereas others sample on a standardized date, such as the predicted date of hatch (see Fig. 1). Given that nesting often coincides with plant growth, the decision about when nests are sampled is potentially very important, as sampling at nest fate results in successful nests being sampled later in the growing season, on average, than failed nests.

Our goal was to evaluate how timing of vegetation surveys influenced the ability to detect the effects of vegetation covariates on nest survival. We addressed this question using simulated data, as well as observational data collected on nesting greater sage‐grouse (*Centrocercus urophasianus*; hereafter sage‐grouse), using average grass height surrounding a nest as an example vegetation covariate. For both simulated and real data sets, we assessed statistical relationships between average grass height covariates and nest survival, and compared results for models that included grass height measured at nest fate with models that included grass height measured at a predicted hatch date. We also compared three different scenarios: (1) grass height had no influence on nest survival, (2) grass height positively influenced nest survival, and (3) grass height negatively influenced nest survival, to further assess the implications of study design for the direction and magnitude of modeled effects. Although we use grass height as an example, the principle we address here applies to any environmental variable that covaries temporally, or is otherwise confounded, with a demographic fate.

## Methods

### Literature review

We performed an informal literature review for ground‐nesting birds nesting in grasslands and shrublands to determine the frequency with which researchers measured vegetation at either the observed date of nest fate, or on a predicted hatch date. We focused exclusively on grassland and shrubland literature as the general hypothesis in these regions is that increased herbaceous ground cover should positively influence nesting success through visual concealment (see review). We only considered publications that assessed the influence of grass height or cover on nest success. We used Google Scholar (http://scholar.google.com) to search for the following key words: nest success, nest survival, grass height, grassland, shrub‐steppe, and we further explored manuscripts that were cited within publications that met the necessary criteria. We excluded publications in which we could not determine the relative timing of the vegetation survey, as well as studies outside of grasslands and shrublands. We report the timing of the vegetation survey (at fate vs. predicted hatch), species, and the general direction of the reported effect of grass height or cover for each publication.

## Simulated Data

### Encounter histories

We developed three different scenarios that varied only in how nest site vegetation (i.e., grass height) influenced the underling survival probability of nests within each data set (i.e., no effect, positive effect, and negative effect). We created 500 replicate encounter histories under each scenario, where each consisted of a maximum of 400 nests that were monitored over an 80‐day nesting season. The nesting season consisted of two phases: (1) nest initiation and (2) nest activity. The nest initiation phase was 40 days long, and we allowed 10 nests to be initiated each day. The nest activity phase lasted the entire 80‐day nesting season, or as long as at least one simulated nest remained active. The length of exposure varied among nests, began with the interval immediately after a nest was initiated, and ended when the nest failed or hatched, whichever came first. Hatch occurred after 37 days of nonfailure. We also included a nest observation term, where each nest had a 0.33 probability of being visited during a given day, and its current nesting state (active/terminated) accurately assigned. The number of nests within each encounter history was therefore variable (${\overset{¯}{x}}_{\mathrm{Ind}}$ = 328.43, SD = 7.55), because some nests failed prior to detection. This allowed us to integrate a realistic degree of stochasticity in the age at which nests entered the sample, which was related to imperfect nest detection. We made the following additional constraints designed to meet model specifications for nest survival models (Dinsmore and Dinsmore 2007). The date of nest discovery was the first observation of an active nest. Nests that failed before an initial observation were censored from the history because they were never discovered. Nests that failed before hatching, but that were observed at least once, were assigned a “last alive” date that coincided with the most recent date the nest was both active and visited by an observer. Failed nests were randomly assigned a “last check” date, which occurred 1–3 days after the nest's true failure date. Nests that hatched were assigned both a last alive and a last check date that were equal to the hatch date of the nest. The average daily nest survival probability (DNS) across all nests in each scenario was constrained to be 0.96, such that DNS was constant among nests within the ‘no‐effect’ scenario and therefore was independent of grass height. For the ‘positive‐effect’ and ‘negative‐effect’ scenarios, we allowed DNS to vary among each nest, *i*, as a function of a linear effect (on the logit scale) of grass height

$$\text{logit}\left({\text{DNS}}_{i}\right)=\left(\beta_{\mathrm{Intercept}}+\beta_{\mathrm{grass}}\times \text{Initial Grass}\;{\text{Height}}_{i}\right)$$

where for the ‘positive‐effect’ scenario, *β*_grass_ = 0.25, and for the ‘negative‐effect’ scenario, *β*_grass_ = −0.25. Initial grass height was the simulated grass height at each nest (described below). *β*_Intercept_ was given as

$$\beta_{\mathrm{Intercept}}=\text{logit}\left(0.96\right).$$

### Simulated grass height covariate

We assigned each nest an initial grass height value, which was calculated as a function of nest initiation date (ID), daily grass growth (Daily Growth), base grass height (Base), and random variation (*ε*).

$$\text{Initial Grass}\;{\text{Height}}_{i}={\text{Base}}_{i}+\text{Daily}\;{\text{Growth}}_{i}\times {\text{ID}}_{i}+\varepsilon_{i}.$$

We estimated grass growth between nest initiation and hatch using grass growth rates given by Hausleitner et al. (2005), who reported that average grass height at sage‐grouse nests changed from 10.0 cm at nest initiation to 15.6 cm at nest hatch, or approximately 0.156 cm/day (mean no. days = 36). We assumed that the mean initiation grass height (10.0 cm) corresponded with the mean nest initiation date (ID = 20), and constrained grass height to grow by 0.156 cm/day, which yielded initial grass heights ranging from 7.04 to 13.11 cm corresponding to nests initiated on days 1–40. We also incorporated a stochastic feature for each nest, where variation was drawn from a normal distribution (${\overset{¯}{x}}_{\varepsilon }$ = 0 cm, SD = 2 cm) and added to the initial grass height measurement. We then allowed grass to grow throughout the nest activity phase, and assigned each nest two additional grass height values: (1) grass height at the date associated with the date last checked (hereafter, fate) and (2) grass height 37 days after nest initiation (hereafter, hatch). These latter values reflected grass heights that would have been recorded if vegetation surveys were conducted at nest fate or on a predicted hatch date, respectively.

## Real Data

We used data collected from nests monitored from 2004 to 2012 in Eureka County, NV, USA (Gibson et al. 2015) to further assess the potential bias in assessing grass height effects associated with timing of vegetation surveys. We measured grass height at all nest sites within 3 days of either the predicted or actual date of hatch. Predicted hatch dates for failed nests were estimated by floating eggs using methods described in detail by Blomberg et al. (2013) and Gibson et al. (2015). Grass height was sampled along 10 m intersecting transects, centered at the nest bowl (Gregg et al. 1994). We used five 20 × 50 cm Daubenmire frames placed along each transect and measured the height (cm) of the nearest representative of live grass to the northeast corner in each frame. We averaged these measurements to estimate mean live grass in the plot associated with each nest. To estimate grass height at nest fate (GH_Fate_), we regressed the measured average grass heights (GH_Survey_) against the ordinal date of the vegetation survey (Date_Survey_) to develop a grass height correction factor (*b*_1_) based on the difference between the ordinal date a nest terminated (Date_Fate_) and Date_Survey_

$${\text{GH}}_{\mathrm{Fate}}={\text{GH}}_{\mathrm{Survey}}-\left({\text{Date}}_{\mathrm{Survey}}-{\text{Date}}_{\mathrm{Fate}}\right)\times b_{1}$$

which yielded predicted grass height measurements as though surveys had been completed immediately after nest fate, assuming a linear growth rate for grass.

### Analysis and model selection

We used RMark (Laake 2013) in R (R Core Team 2012) to call the nest survival module in the program MARK (White and Burnham 1999), which we used to estimate the effect size of grass height on daily nest survival for each simulated and real data set. For both simulated and real data sets, we considered three models: (1) constant DNS (null model); (2) DNS varied by the average grass height on the date of recorded nest fate; and (3) DNS varied by the average grass height on the predicted hatch date. We used an information theoretic approach to evaluate support for candidate models (Burnham and Anderson 2002), and considered covariate effects to be meaningful if 85% confidence intervals of *β* coefficients did not overlap 0.0 (Arnold 2010). For the simulated scenarios, we used the mean and standard deviation of parameter estimates, as well as mean ΔAIC_*c*_ and AIC_*c*_ model weights (*w*_*i*_). We focus our assessment of results on the extent to which simulated scenarios deviate from our known effect sizes, and in the case of the real data, how our inferences related to grass height effects changed depending on how we incorporated measures of nest vegetation.

## Results

We reviewed 28 publications involving 19 species, and found that 22 (~79% of studies) sampled vegetation relative to nest fate, whereas six sampled vegetation relative to a predicted hatch date (Table 1). Some publications reported independent effects for multiple species, and we report effects relative to the total number of analyses (*n* = 45) across all publications. Of the analyses based on vegetation data sampled at nest fate, 25 (~74%) of studies reported a positive effect of grass height or cover on nest survival, while nine (~26%) analyses lacked support for an effect of grass height or cover. Of the analyses based on vegetation data sampled at predicted hatch date, two (~33%) reported a positive effect of grass height or cover on nest survival, while four (~67%) analyses lacked support for an effect of grass height or cover (Fig. 1, Table 1).

**Table 1:** Summary of literature review assessing variation in study design for studies assessing the influence of nest site grass height or cover on nest survival for grassland or shrubland bird species. Two common survey protocols included sampling nest vegetation at nest fate (i.e., hatch or failure) or on a predicted hatch date, and publications reported positive and no support for an effect of grass. For studies that considered multiple species of bird, values in ( ) represent the number of species reported to have the specified relationship between grass height and nest survival

Species	Timing of survey	Direction of effect	Source
Grasshopper sparrow (*Ammodramus savannarum*)	Fate	Positive	Lyons (2013)
Clay‐colored sparrow (*Spizella pallida*), Savannah sparrow (*Passerculus sandwichensis*), and bobolink (*Dolichonyx oryzivorus*)	Fate	Positive (1), no support (2)	Kerns et al. (2010)
Clay‐colored sparrow (*Spizella pallida*), Savannah sparrow (*Passerculus sandwichensis*), and bobolink (*Dolichonyx oryzivorus*)	Fate	Positive (3)	Winter et al. (2005)
Sprague's Pipit (*Anthus spragueii*), Savannah Sparrow (*Passerculus sandwichensis*), Baird's Sparrow (*Ammodramus bairdii*), Chestnut‐collared Longspur (*Calcarius ornatus*), and Western Meadowlark (*Sturnellaneglecta*)	Fate	Positive (4), no support (1)	Davis (2005)
Brewer's Sparrows (*Spizella breweri*), Horned Lark (*Eremophila alpestris*), Sage Thrasher (*Oreoscoptes montanus*), Savannah Sparrow (*Passerculus sandwichensis*), Vesper Sparrow (*Pooecetes gramineus*), and Western Meadowlarks (*Sturnella neglecta*)	Fate	Positive (2), no support (4)	Vander Haegen et al. (2015)
Brewer's Sparrows (*Spizella breweri*), Lark Sparrows (*Chondestes grammacus*), Vesper Sparrows (*Pooecetes gramineus*), and Western Meadowlarks (*Sturnella neglecta*)	Fate	Positive	Knight et al. (2014)
Vesper sparrow (*Pooecetes gramineus*)	Fate	Positive	Sadoti et al. (2014)
Greater Prairie Chicken (*Tympanuchus cupido*)	Fate	Positive	McKee et al. (1998)
Greater Prairie Chicken (*Tympanuchus cupido*)	Fate	Positive	McNew et al. (2014)
Greater Sage‐grouse (*Centrocercus urophasianus*)	Fate	Positive	Doherty et al. (2014)
Greater Sage‐grouse (*Centrocercus urophasianus*)	Fate	Positive	Doherty et al. (2011)
Greater Sage‐grouse (*Centrocercus urophasianus*)	Fate	Positive	Coates & Delehanty (2010)
Greater Sage‐grouse (*Centrocercus urophasianus*)	Fate	No support	Kolada et al. (2009)
Greater Sage‐grouse (*Centrocercus urophasianus*)	Fate	Positive	Lockyer et al. (2015)
Greater Sage‐grouse (*Centrocercus urophasianus*)	Fate	Positive	Popham & Gutierrez (2003)
Greater Sage‐grouse (*Centrocercus urophasianus*)	Fate	Positive	Wing (2014)
Greater Sage‐grouse (*Centrocercus urophasianus*)	Fate	Positive	Kaczor et al. (2011)
Greater Sage‐grouse (*Centrocercus urophasianus*)	Fate	Positive	Bell (2011)
Greater Sage‐grouse (*Centrocercus urophasianus*)	Fate	Positive	Rebholz (2008)
Gunnison Sage‐grouse (*Centrocercus minimus*)	Fate	Positive	Stanley et al. (2015)
Lesser Prairie Chicken (*Tympanuchus pallidicinctus*)	Fate	Positive	Pitman et al. (2005)
Greater Sage‐grouse (*Centrocercus urophasianus*)	Predicted Hatch	No support	Gibson (2015)
Greater Sage‐grouse (*Centrocercus urophasianus*)	Predicted Hatch	Positive	Gregg et al. (1994)
Greater Sage‐grouse (*Centrocercus urophasianus*)	Predicted Hatch	No support	Davis et al. (2014)
Greater Sage‐grouse (*Centrocercus urophasianus*)	Predicted Hatch	Positive	Sveum et al. (1998)
Lesser Prairie Chicken (*Tympanuchus pallidicinctus*)	Predicted Hatch	No support	Davis (2009)
Northern Bobwhite (*Colinus virginianus*)	Fate	No support	Rader et al. (2007)
Long‐billed Curlew (*Numenius americanus*)	Predicted Hatch	No support	Gregory et al. (2011)

### Simulated data

**Figure 2:**
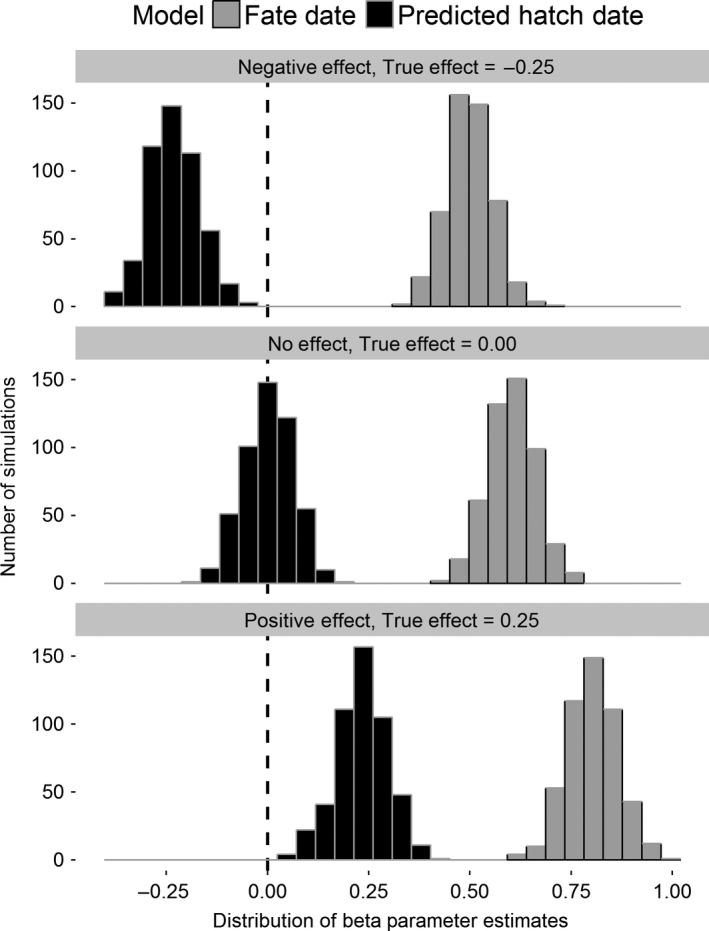
The distribution of parameter coefficient estimates for nest survival models that differed based on whether vegetation was measured on a predicted nest hatch date (black) or on the date of nest fate (gray). Three scenarios were considered where grass height reduced nest survival (top panel), had no influence on nest survival (center panel) and where grass height positively influenced nest survival (bottom panel). Each scenario was evaluated using 500 iterations of simulated nest survival data.

Our simulations suggested that measuring grass height at nest fate resulted in effect sizes that were positively biased relative to true effects. Under the negative effect of grass height on nest survival scenario, measurement at fate produced a positive effect (${\overset{¯}{\beta }}_{\mathrm{fate}}$ = 0.50, SD = 0.06) whereas measurement at hatch produced a negative effect that was close to the true effect (${\overset{¯}{\beta }}_{\mathrm{hatch}}$ = −0.23, SD = 0.06). Under the no‐effect scenario, measurement at fate also produced a positive effect (${\overset{¯}{\beta }}_{\mathrm{fate}}$ = 0.60, SD = 0.06) whereas measurement at hatch correctly predicted no effect (${\overset{¯}{\beta }}_{\mathrm{hatch}}$ = 0.00, SD = 0.06). Under the positive‐effect scenario, measurement at fate correctly identified a positive effect, but the effect magnitude was more than three times greater (${\overset{¯}{\beta }}_{\mathrm{fate}}$ = 0.80, SD = 0.06) than the true effect size, which again was correctly approximated by measurement at hatch (${\overset{¯}{\beta }}_{\mathrm{hatch}}$ = 0.23, SD = 0.06); Fig. 2).

In addition to producing biased effect sizes, models based on measurement at fate were better supported in model selection, substantially outcompeting the grass height at hatch models (which accurately described the true demographic mechanism in the data; Tables 2, 3, 4). Thus, our simulations indicate that models of covariate effects based on measurement at fate will be favored as predictive based on established model selection procedures (Burnham and Anderson 2002), even in situations where no effect of the covariate exists in nature. Furthermore, our results suggest that measuring grass height at nest fate may result in a false‐positive reporting of effects when no effect exists (Fig. 2).

**Table 2:** Summary of the performance of nest survival models in Program MARK used to assess the influence of timing of vegetation surveys at nest sites on nest survival. Results are based on 500 iterations, each with unique encounter histories in which the underlying daily nest survival was positively influenced by grass height. All reported results are average values across all iterations. We do not report the average model deviance as it would be uninformative

Model^a^	ΔAIC_*c*_	*w*_*i*_	No. par	*β*	SE
Grass Height_Fate_	0.01	1.00	2	0.80	0.07
Grass Height_Hatch_	80.78	0.00	1	0.23	0.07
Constant	91.39	0.00	1	3.28	0.07

a Model selection notation follows Burnham and Anderson (2002). All models included an intercept‐term. Grass Height_Fate_ represents models that included a covariate based on the simulated grass height at a nest on the date the nest's fate was assigned. Grass Height_Hatch_ represents models that included a covariate based on the simulated grass height at a nest on the date a nest hatched, or was supposed to hatch, if unsuccessful. Constant represents the null, or intercept‐only model.

**Table 3:** Summary of the performance of nest survival models in Program MARK used to assess the influence of timing of vegetation surveys at nest sites on nest survival. Results are based on 500 iterations, each with unique encounter histories in which the underlying daily nest survival was negatively influenced by grass height. All reported results are average values across all iterations. We do not report the average model deviance as it would be uninformative

Model^a^	ΔAIC_*c*_	*w*_*i*_	No. par	*β*	SE
Grass Height_Fate_	0.04	0.99	2	0.50	0.07
Grass Height_Hatch_	39.32	0.00	2	−0.23	0.07
Constant	49.73	0.00	1	3.28	0.07

a Model selection notation follows Burnham and Anderson (2002). All models included an intercept‐term. Grass Height_Fate_ represents models that included a covariate based on the simulated grass height at a nest on the date the nest's fate was assigned. Grass Height_Hatch_ represents models that included a covariate based on the simulated grass height at a nest on the date a nest hatched, or was supposed to hatch, if unsuccessful. Constant represents the null, or intercept‐only model.

**Table 4:** Summary of the performance of nest survival models in Program MARK used to assess the influence of timing of vegetation surveys at nest sites on nest survival. Results are based on 500 iterations, each with unique encounter histories in which the underlying daily nest survival was not influenced by grass height. All reported results are average values across all iterations. We do not report the average model deviance as it would be uninformative

Model^a^	ΔAIC_*c*_	*w*_*i*_	No. par	*β*	SE
Grass Height_Fate_	0.00	1.00	2	0.60	0.07
Constant	72.90	0.00	1	3.28	0.07
Grass Height_Hatch_	74.05	0.00	2	0.00	0.07

a Model selection notation follows Burnham and Anderson (2002). All models included an intercept‐term. Grass Height_Fate_ represents models that included a covariate based on the simulated grass height at a nest on the date the nest's fate was assigned. Grass Height_Hatch_ represents models that included a covariate based on the simulated grass height at a nest on the date a nest hatched, or was supposed to hatch, if unsuccessful. Constant represents the null, or intercept‐only model.

Although we found that parameter coefficients were biased relative to timing of vegetation sampling, mean estimates of daily nest survival (DNS) were identical for models that measured vegetation at fate (${\bar{\text{DNS}}}_{\mathrm{fate}}$ = 0.964, SE = 0.002) and hatch (${\bar{\text{DNS}}}_{\mathrm{hatch}}$ = 0.964, SE = 0.002), and also matched our simulated conditions (DNS = 0.96). Thus bias associated with measurement error was associated with parameter coefficients and predicted DNS for a specific covariate value, but estimates of nest survival for the sample as a whole were unaffected. Or in other words, the mean estimate (i.e., the intercept) of nest survival was not influenced by the confounding effect of measurement error, as the sampling bias was solely attributed to the specified parameter coefficient (i.e., slope).

### Real data

**Figure 3:**
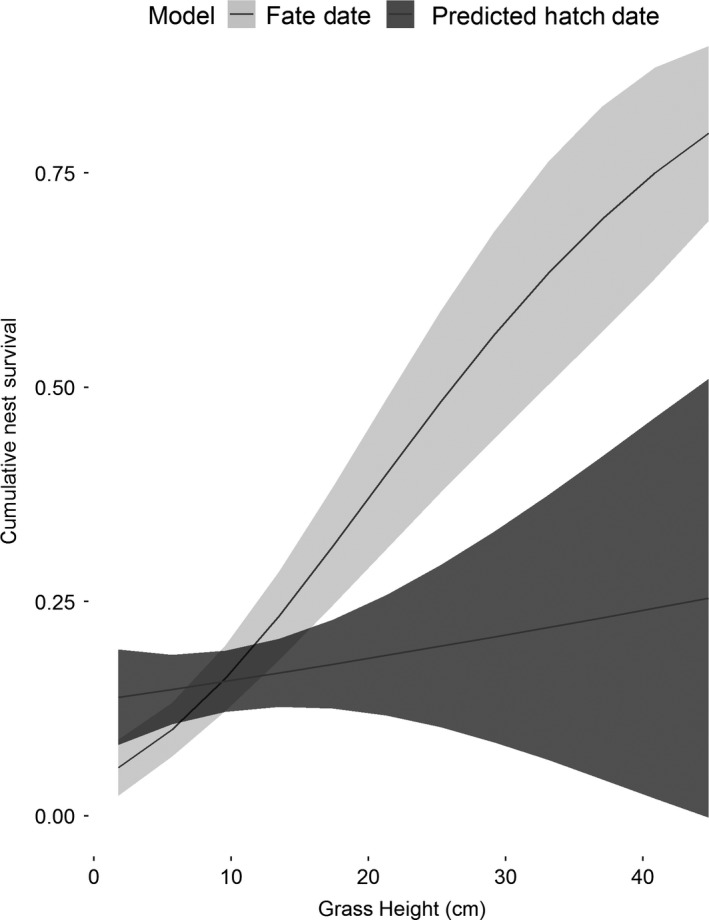
Estimated probability of cumulative nest survival relative to the average grass height within 100 m^2^ of a nest, where grass was measured on the predicted hatch date of a nest (gray line, dark gray ribbon) or was predicted based on the date of nest fate (black line, light gray ribbon) for Greater Sage‐Grouse in Eureka, Nevada, USA from 2004–2012. Predicted grass height at fate was estimated by regressing average grass heights against ordinal dates of vegetation surveys to correct grass height measurements based on daily growth rates.

Model selection and parameter estimates from the sage‐grouse nest survival analysis based on real data mirrored results from the simulated data. These results also suggested that the derived GH_fate_ variable produced effect sizes that were greater (*β*_fate_ = 0.47; SE_fate_ = 0.09) than that of the GH_hatch_ variable (*β*_hatch_ = 0.06; SE_hatch_ = 0.06). Similar to the simulated results, the GH_fate_ variable was also better supported in model selection, outcompeting the GH_hatch_ model by more than 30 AIC units and receiving all of the AIC model weight (Table 5). Interpretation of grass height influence on sage‐grouse nest survival was also different between the two metrics; the GH_fate_ variable suggested a strong positive effect of grass height on sage‐grouse nest survival, whereas GH_hatch_ suggested only a very weak effect (Fig. 3).

**Table 5:** Performance of nest survival models in Program MARK used to assess the influence of timing of vegetation surveys at nest sites on Greater sage‐grouse nest survival in Eureka County, NV, 2004–2012

Model^a^	ΔAIC_*c*_	AIC_*c*_ *w*_*i*_	No. par	Dev.	*β*	SE
Grass Height_Fate_	0.00	1.00	2	1478.72	0.47	0.09
Constant	31.61	0.00	1	1512.34	2.97	0.06
Grass Height_Hatch_	32.59	0.00	2	1511.31	0.06	0.06

a Model selection notation follows Burnham and Anderson (2002). All models included an intercept‐term. Grass Height_Hatch_ represents a model that included a covariate based on the measured grass height at a nest on the date a nest hatched, or was supposed to hatch, if unsuccessful. Grass Height_Fate_ represents a model that included a covariate based on the estimated grass height at a nest on the date the nest's fate was assigned, which was derived from Grass Height_Hatch_ and estimated daily grass growth. Constant represents the null, or intercept‐only model.

## Discussion

Our results provide multiple lines of evidence that confounding between plant phonology and demographic processes can have important implications when evaluating vegetation effects on vital rates, in our case nest survival. We found that studies of grassland and shrubland birds predominantly used nest site vegetation metrics sampled at the time of nest fate, which was more likely to yield a positive effect of grass height or cover when compared to data collected on a standardized date, such as predicted hatch. Our simulations, and evidence from real data, also show that grass height measurements recorded at nest fate produced effect sizes that were biased high relative to the true effect of grass height, and in some cases, this bias was sufficient to change the overall direction of the effect as well as its magnitude. These results are undoubtedly related to the inherent confounding between grass growth and timing of fate for failed versus successful nests; the fate of successful nests occurs inherently later in the season, therefore vegetation biomass will increase prior to sampling for successful nests when compared with unsuccessful nests, which fail and are sampled earlier. Most notably, models based on vegetation data collected at nest fate were more parsimonious (i.e., lower AIC value) relative to models based on vegetation data collected at hatch. This was completely an artifact, however, driven by the aforementioned confounding between grass growth and nest fate, and the increase in explanatory power was related to the covariate accounting for the confounding between fate and the value of the covariate introduced into the model by sampling at nest fate. Sampling nests on a standardized date that is also biologically meaningful, such as the predicted hatch date, appear to overcome these issues and produce robust estimates of vegetation effects on nest survival.

Although we have focused on grass height and nest survival, confounding among plant phenology and demographic process is clearly a more general issue because of the role that vegetation structure and composition play in the study of animal ecology from both basic and applied perspectives (e.g., Fisher and Davis 2011; Germain and Arcese 2014). Key to our results is an inherent confounding between the timing of nest fate and the timing of vegetation sampling when measured at fate. Other demographic traits, such as age‐specific survival or breeding probabilities, could be similarly affected if careful consideration is not given to potential confounding factors during study design and data analysis. This would include both the timing of vegetation sampling, and the temporal resolution of the demographic estimate, as well as the potential correspondence of these two measures. For example, age‐ or stage‐specific survival is commonly expressed as a probability value that reflects the likelihood an individual will survive a given time interval (e.g., a week, month, year, etc.). If vegetation is sampled at a finer time interval (e.g., weekly), and then is applied as a covariate to an interval that is more coarse (e.g., monthly survival probability), and if vegetation is likely to change due to growth or senescence throughout the monthly interval, mean vegetation measures may differ for individuals that die early in the month compared to those that survive the duration of the month. In this case, the inherent confounding between vegetation and demographics can be resolved by estimating survival probability at a temporal resolution that matches that of the vegetation sampling (e.g., a weekly survival probability), and by including vegetation measures as time‐varying covariates during data analysis (Bonner et al. 2010).

Increased grass height or cover is correlated with reduced visibility (Carlyle et al. 2010), and we agree that a positive association between grass biomass and nest survival is intuitive, especially for ground‐nesting species. In our positive‐effect simulations, grass height measured at nest fate correctly identified the positive association between grass and nest survival. However, the magnitude of the effect, as evidenced by the modeled parameter coefficient, was inflated relative to the true effect. Depending on study objectives, the magnitude of an effect may be as important for biological interpretation as the fact that the effect exists in the first place. This consideration may be particularly important to identify conservation guidelines or targets for vegetation management (e.g., Connelly et al. 2000; Hagen et al. 2004) because modeled parameter coefficients can be used to identify management thresholds, and will be inflated in a scenario of positive sampling bias (Fig. 3). Additionally, as conservation plans are often inadequately funded, the appropriation of resources toward management objectives based on habitat metric that has not reliably been demonstrated to improve reproductive performance has additional consequences as it reduces the amount of resources available for more meaningful restoration efforts. We also appreciate the rationale for measuring vegetation at nest fate; nests presumably fail because of conditions that are present at the time of failure (e.g., vegetation failing to conceal a nest from a predator) and so measuring those conditions are somewhat intuitive. This approach, however, cannot disentangle the confounding between the timing of vegetation sampling and nest fate from the true demographic mechanism associated with vegetation concealment, as our simulations demonstrate.

When designing future research, we recommend that investigators carefully consider confounding between plant phenology and their demographic rate of interest, and conduct vegetation sampling accordingly. In situations where vegetation sampling was conducted at nest fate, measurements can be date‐corrected to remove the potential confounding between the timing of nest fate and vegetation measurement. This can be accomplished by regressing the vegetation measurement on the ordinal date of the survey, and using the model residuals as date‐corrected estimates. Alternatively, we have outlined above an approach to predict grass height at nest fate based on measured grass height on a predicted hatch date. A slight modification could also be used to forecast vegetation measurements based on system‐specific growth rates and timing of surveys at fate relative to a predicted hatch date. This latter approach also has the advantage of creating date‐corrected vegetation measurements that fall within the same range as values likely to be measured in the field, as opposed to the residual‐based approach which would yield both positive and negative values relative to the modeled regression line. Although these two approaches are not perfect, they should serve to disentangle confounding between plant growth and timing of nest failure, effectively removing a source of sampling bias from the data. Both approaches assume that vegetation growth is linear with respect to ordinal date, however nonlinear growth could be easily incorporated using quadratic effect terms. Lastly, we speculate that future advancements in automated aerial technology, spatial imaging resolution and classification may allow researchers to quantify fine‐scale habitat characteristics while nests are active without excessive disturbance, effectively disentangling plant growth and timing of nest fate (Connelly et al. 2000; Drever et al. 2015).
